# Use of roxadustat (FG-4592) in ruxolitinib-treatment-related anemia of two myelofibrosis patients

**DOI:** 10.1007/s00277-022-04997-3

**Published:** 2023-03-13

**Authors:** Kai Ding, Zhaoyun Liu, Yue Ren, Hui Liu, Rong Fu

**Affiliations:** Department of Hematology, Tianjin Medical University General Hospital, Tianjin, 300052 People’s Republic of China

Dear Editor,

We report two myelofibrosis (MF) cases with continuously transfusion-dependent anemia after ruxolitinib treatment, which was corrected by roxadustat (FG-4592), a novel, orally bioavailable, heterocyclic small molecule that reversibly inhibits hypoxia-inducible factor prolyl hydroxylase (HIF-PH) enzymes and activates HIF and the transcription of HIF-responsive genes, including endogenous erythropoietin (EPO) [1]. These cases suggest a new choice in MF-related anemia and ruxolitinib-induced anemia and warrant further clinical trials and laboratory studies.

**Fig. 1:**
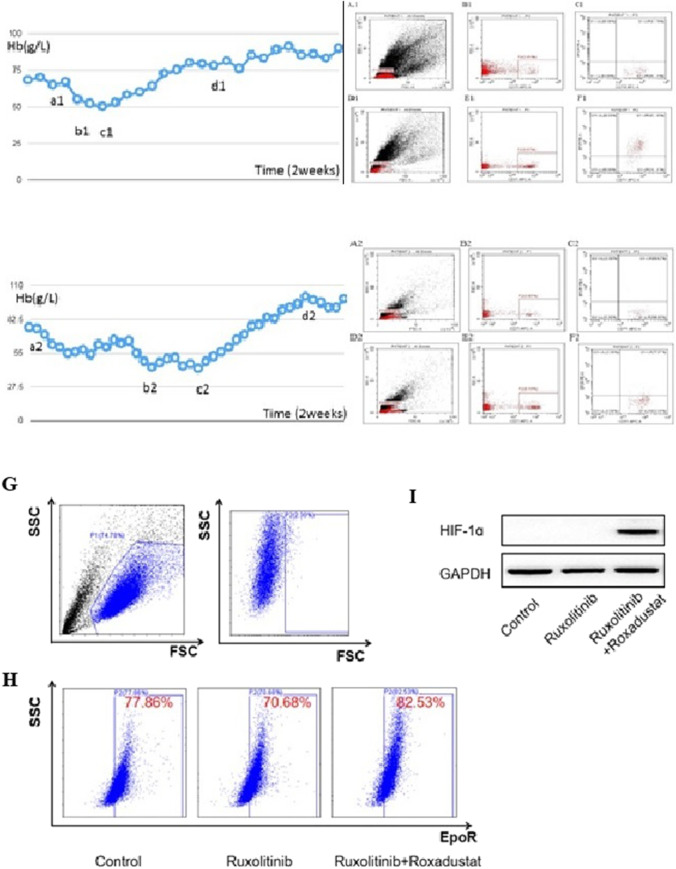
Hemoglobin level changes and CD71 and EpoR expression in bone marrow mononuclear cells of myelofibrosis patients, changes in EpoR and HIF-1ɑ expression following ruxolitinib and roxadustat treatment of K562 cells. Patient 1: changes in hemoglobin level. **a1** Ruxolitinib 20 mg twice daily, EPO 6000 U every other day. **b1** Ruxolitinib 10 mg twice daily, EPO 6000 U every other day, android 80 mg twice daily. **c1** Ruxolitinib 10 mg twice daily, roxadustat 100 mg three times per week, android 80 mg twice daily. **d1** Ruxolitinib 20 mg twice daily, roxadustat 100 mg three times per week. Patient 2: changes in hemoglobin level. **a2** Ruxolitinib 20 mg twice daily, andriol 40 mg twice daily, dexamethasone 0.75 mg daily, EPO 6000 U every other day. **b2** Blood transfusion-dependent, drugs unchanged. **c2** Ruxolitinib 20 mg twice daily, andriol 40 mg twice daily, dexamethasone 0.75 mg daily, roxadustat 100 mg three times per week. **d2** Ruxolitinib 20 mg twice daily, roxadustat 100 mg three times per week. Patients 1 and 2: expression levels of CD71 and EpoR in bone marrow mononuclear cells before (panels **B** and **C**) and after (panels **E** and **F**) roxadustat treatment. Panels **B** and **E**: percentage of CD71^+^ cells. Panels **C** and **F**: percentage of CD71^+^EpoR^+^ cells. **G** Flow cytometry gating strategy. **H** Controls included dimethyl sulfoxide (DMSO) and ruxolitinib treatments alone; K562 cells were treated with ruxolitinib for 48 h followed by roxadustat for 48 h. Expression of EpoR on the surface of K562 cells treated with different treatments. **I** HIF-1ɑ expression in K562 cells treated with different treatments, determined using western blotting

The patients’ information and primary therapeutic regimes are shown in Table 1. The first patient was admitted owing to progressive constitutional symptoms and massive splenomegaly a year and a half ago, and the diagnosis of the first case shifted to post-ET MF with a DIPSS risk of intermediate-2. The therapy regimen and hemoglobin levels are shown in Fig. 1 a1–d1. After 6 months of treatment with roxadustat, the patient’s hemoglobin was 83 g/L. The spleen volume reduced to 2 cm below the costal margin; the mutation ratio of JAK2 decreased to 33.32%. For the second case, in the preceding 2 years, symptoms of anemia and splenomegaly deteriorated, and hemoglobin level dropped from 80–90 to 70–80 g/L. The therapy regimen and hemoglobin levels are shown in Fig. 1 a2–d2. The patient was followed up for 9 months after roxadustat administration; his CBC remained stable without MF-related symptoms. Using flow cytometry, the percentage of erythroblasts in patients’ bone marrow was increased from 2.81 to 9.87% (case 1), 3.85 to 6.18% (case 2). And EpoR expression on erythroblast membrane was increased from 0.79 to 85.99% (case 1) (Fig. 1 A1–F1), 0.62 to 7.97% (case 2) (Fig. 1 A2–F2).

**Table 1:** Clinical baseline data of myelofibrosis patients

	Case 1	Case 2
Age	65	61
Gender	M	M
Early diagnosis	ET for 6 years, DIPSS risk of intermediate-2	primary MF, DIPSS risk of intermediate-2
WBC (10^9^/L)	24.42	4.48
Platelet (10^9^/L)	595	334
RBC (10^9^/L)	2.11	2.40
Hb (g/L)	68	76
Folic acid (ng/mL)	4.31	2.77
vitamin B12 (pg/mL)	1272.88	454.19
ferritin (ng/mL)	808.22	1039.02
EPO (IU/L)	22.70	201.3
Bone marrow biopsy	Trilineage hypercellularity and MF (3 +)	Trilineage hypercellularity without blast cells and MF (4 +)
Primary therapeutic regimen	hydroxyurea 1.0 g twice daily and aspirin (100 mg daily)	Ruxolitinib 20 mg twice daily, andriol 40 mg twice daily, dexamethasone 0.75 mg daily, and EPO 6000 U every other day
Gene sequencing	*JAK2* V617 mutation 96.80%; *MPL*, *CARL*, and *MDS*-associated gene mutations (-)	*CALR* mutation 38.70%, *MPL*, *JAK2* V617F, and *MDS*-associated gene mutations (-)

Given the vital role of the JAK-STAT pathway in erythropoietin-mediated signaling, anemia is a prominent toxicity of the archetypal JAK inhibitor, ruxolitinib [2]. Roxadustat improves iron regulation by modulating hepcidin levels [3]. Hypoxic conditions enhance EPO expression, RBC mass, and hemoglobin levels via the coordinated expression of EPO and genes responsible for other aspects of erythropoiesis, including iron absorption, transport, storage, metabolism, and heme synthesis, all of which contribute to the production of new RBCs [1, 4, 5]. Many genes involved in this coordinated erythropoietic response to hypoxia are regulated by HIF. Both our cases experienced MF-related anemia that exacerbated after ruxolitinib treatment, and both responded poorly to EPO and androgen treatment. After roxadustat treatment, ruxolitinib-related anemia was relieved in both patients. Their hemoglobin levels were even higher than those prior to ruxolitinib treatment. Both patients experienced RBC transfusion dependence after ruxolitinib treatment, and their ferritin levels increased to 808.22 and 1039.02 ng/mL. After the increase in hemoglobin levels following roxadustat treatment, ferritin levels decreased to 35.83 and 155.90 ng/mL. Roxadustat can modulate iron utilization in the erythropoietic process. The percentage of erythroblasts in the bone marrow and EpoR expression on erythroblast membrane improved in both patients after roxadustat treatment (Fig. 1 A1–F1, A2–F2). Our primary experiments in K562 cells demonstrated that roxadustat could upregulate HIF-1ɑ and EpoR expression (Fig. 1G–I), which was suppressed by ruxolitinib, illustrating another probable mechanism for the recovery of anemia in these two patients.
